# Epigenetic Control of Reprogramming and Cellular Differentiation

**DOI:** 10.1155/2012/538639

**Published:** 2012-09-17

**Authors:** Lucia Latella, Daniela Palacios, Sonia Forcales, Pier Lorenzo Puri

**Affiliations:** ^1^Dulbecco Telethon Institute (DTI), IRCCS Fondazione Santa Lucia and European Brain Research Institute, 00143 Rome, Italy; ^2^National Research Council, Institute of Translational Pharmacology, via Fosso del Cavaliere 100, 00133 Rome, Italy; ^3^Institute of Predictive and Personalized Medicine of Cancer (IMPPC), 08916 Badalona, Spain; ^4^Sanford-Burnham Institute for Medical Research, La Jolla, CA 92037, USA

Recently, numerous interdisciplinary approaches that include integration of bioinformatics, epigenetics, and molecular and cellular biology have started to shed light on the complex dynamics that regulate cellular processes such as cell “stemness,” reprogramming, and differentiation. From the sequencing of the first complete genomes to the recent high-throughput genomic and proteomic approaches, a huge amount of information has been generated to improve our understanding of how the eukaryotic genome works to activate and coordinate specific programs of gene expression.

The focus of this review is to provide an overview of the epigenetic regulatory mechanisms controlling crucial cellular fate decisions, in particular during differentiation and reprogramming. A special emphasis has been given to skeletal myogenesis, as it provides an amenable model for in vitro and in vivo studies.

Y. Ito et al. in “*A system approach and skeletal myogenesis*” provide an overview of the most common approaches currently used to address transcriptional regulation. They discuss deep sequencing and microarray-based methods, cell-based high throughput assays, and the generation of *in situ* gene expression databases as potent tools to dissect complex networks of gene expression. Using skeletal muscle as a paradigm, they highlight the main discoveries obtained through the different methods and underline the advantages of an integrated approach to obtain a full picture of cellular differentiation. 

An increasing number of studies suggest an epigenetic component in the development of many human diseases. In the review article “*Epigenetic alterations in muscular disorders,*” Dr. C. Lanzuolo discusses how altered epigenetic mechanisms contribute to human pathology and in particular to the development and progression of neuromuscular disorders. Muscular dystrophies arise from either mutations of proteins that play a role in the integrity of the sarcolemma—the plasma membrane that wraps the myofibers in skeletal muscles—or are due to a dysfunction within the cell nucleus. Dr. C. Lanzuolo focuses on the so-called nuclear muscular dystrophies and she analyzes the possible contribution of an important group of epigenetic modifiers, the polycomb-group (PcG) complexes, to the pathogenesis of these diseases. The dissection of the aberrant epigenetic profiles associated to several human pathologies has contributed to the design of experimental therapies with epigenetic drugs.

The review “*Tackling skeletal muscle cells epigenome in the next-generation sequencing era*” by R. Fittipaldi and G. Caretti points to the significance of genome-wide study of the epigenetic modifications and the transcription factors that are activated during skeletal muscle differentiation. The authors discuss on the new genome-wide technologies to monitor the epigenetic landscape that characterizes myoblast-to-myotube transition in order to obtain innovative information on molecular mechanisms orchestrating biological processes, such myogenic differentiation.

The fine regulation of a myogenic gene such as *myogenin* is discussed in “*Turning on myogenin in muscle: A paradigm for understanding mechanisms of tissue-specific gene expression*” by H. Faralli and F. J. Dilworth. Transcription factors, cofactors, and epigenetic modifications contribute to regulate muscle-specific gene expression during development. In particular, the authors highlight the fact that a tissue-specific factors such as MyoD cooperate with more ubiquitously expressed proteins (Six4, Mef2, and Pbx1) to establish a transcriptionally permissive environment to ensure proper spatiotemporal *myogenin* expression.

The current challenge of epigenetics is how to analyse and extract biological knowledge from the large volumes of data produced with new high-throughput technologies. In this regard, the paper of S. Althammer et al., “*Predictive models of gene regulation from high-throughput epigenomics data*” describes a computational framework using integrative tools and machine learning algorithms that facilitate the systematic analysis of high-throughput sequencing epigenetic data for integrative studies. Their analysis shows a different epigenetic code of expression for intron-less and intron-containing genes, with more prominent differences for genes with low GC content (LGC) in their promoter, showing for instance that at the promoter regions of LGC intron-less genes, H3K36me3 and H3K4me1 are the most informative marks while in polyadenylation sites of expressed genes, the H3K36me3 signal is much weaker than in intron-containing genes. Based on epigenetic data, their model predicts very strongly gene expression between two conditions; therefore, this approach can be applied to study gene expression in different developmental stages, disease states, or treatments.

Of tremendous relevance for its therapeutic potential, is the possibility to manipulate cell identity. In the review titled “*The stability of the induced epigenetic programs*”, M. J. Barrero discusses the epigenetic changes that occur during differentiation and the way back upon cellular reprogramming emphasizing on the thin regulation of pluripotent and lineage-specific genes expression to ensure a correct differentiation during development and in adult life.

“*The epigenetic regulation of B lymphocyte differentiation, transdifferentiation, and reprogramming”* by B. Barneda-Zahonero et al. reviews the hierarchical network of transcription factors that mediate the epigenetic signature regulating the transcriptome during B cell development. B lymphocytes are highly specialized terminally differentiated cells responsible for generating high-affinity antibodies, providing the humoral immunity against pathogens. Every day the human body generates millions of B lymphocytes from precursor hematopoietic stem cells by a differentiation process that is a tightly regulated. This multistep differentiation process, vital to immunity, combines the successive expression of specific transcriptional factors and associated epigenetic changes to restrict the developmental potential of lymphoid progenitors to the B cell pathway by repressing B-lineage-inappropriate genes, while simultaneously promoting B cell development by activating B-lymphoid-specific genes. 

*Lucia Latella* *Lucia Latella* *Daniela Palacios* *Daniela Palacios* *Sonia Forcales* *Sonia Forcales* *Pier Lorenzo Puri* *Pier Lorenzo Puri*

